# Helmut Gadner: A Charismatic Leader, Visionary, and Pioneer in Pediatric Oncology

**DOI:** 10.7759/cureus.68610

**Published:** 2024-09-04

**Authors:** Milen Minkov, Leo Kager

**Affiliations:** 1 Studies and Statistics for Integrated Research and Projects (S2IRP), St. Anna Children's Cancer Research Institute (CCRI), Vienna, AUT; 2 Research Center for Childhealth, Growth, and Development, Sigmund Freud Private University, Vienna, AUT; 3 Department of Pediatrics and Adolescent Medicine , Division of Pediatric Oncology, Johannes Kepler University, Linz, AUT; 4 Department of Pediatric Hematology/Oncology, St. Anna Children's Cancer Research Institute (CCRI), Vienna, AUT; 5 Department of Pediatrics and Adolescent Medicine, Medical University of Vienna, St. Anna Children's Hospital, Vienna, AUT

**Keywords:** pediatric cancer, pediatric leukemia, medical innovation, medical stories, biographies, historical vignette

## Abstract

Helmut Gadner, a world-famous pediatric hematologist-oncologist, dedicated his professional life to researching pediatric cancer and advancing care for affected children, adolescents, and their families. Starting his career in the team of Hansjörg Riehm, the father of the ever-most successful BFM (abbreviation of the city names of the founding institutions, Berlin-Frankfurt-Münster) treatment concept of pediatric leukemia, the young Dr. Gadner became a passionate soldier in the fight against pediatric cancer. From 1980 to 2010, he served as a medical director of St. Anna Children's Hospital in Vienna, Austria. Soon after taking this highly responsible position, he realized that improving patient care requires rigorous research from bench to bedside. In 1988, he and others founded St. Anna Children's Cancer Research Institute (St. Anna CCRI), a testament to his visionary spirit, which he headed until his retirement in 2012. His thoughtful leadership and vision were instrumental in establishing St. Anna Children's Hospital and St. Anna CCRI as a worldwide acclaimed center for researching, diagnosing, and treating children and adolescents with cancer within the environment of national and international collaborations and clinical trials. Dr. Gadner's enduring legacy, St. Anna CCRI, recently celebrated its 35^th^ anniversary, operating 14 research groups actively engaged in global collaboration and a wide range of research fields, from basic molecular research to prospective international clinical trials.

Helmut Gadner was chair of the Austrian National Working Group for Pediatric Hematology and Oncology (AGPHO) for many years. The outstanding survival of children with acute lymphoblastic leukemia in Austria, placing pediatric oncology in this small country at the top of Europe and the world, is the best evidence of his dedicated and perseverant leadership in this position. Dr. Gadner's lifetime achievements as a scientist, physician, mentor, and leader have received many national and international awards, and herein we try to provide a summary.

## Introduction and background

Early life, education, and personal merits

Helmut Gadner was born in 1940 in Bolzano (South Tyrol, Italy). He grew up bilingual (German and Italian) in his birthplace, where he attended Franciscan high school and graduated in 1959 [1]. Born and raised in the magnificent Dolomites, Helmut Gadner has been an enthusiastic mountaineer since childhood. He climbed countless peaks in his homeland and some famous mountains abroad with his younger brother. He retained this passion throughout his life and passed it on to his children and grandchildren. Helmut was also an ardent biker, and riding an exceptional mountain bike with the iconic Cannondale "lefty fork" underscores his unique sense of perfection.

Helmut Gadner studied medicine in Vienna (Austria) and Freiburg (Germany). In 1966, he completed the Doctor of Medicine (M.D.) program at Vienna University. In 1969, after three years in which he gathered his first medical experiences at a local hospital in Vinschgau, Italy, completed his military service, and attended some additional pediatric medical training programs in Modena and Bologna, he started his residency training as a pediatrician at the Freie Universität/Charité in West Berlin [1]. Around that time, he met and soon married his wonderful wife Waltraud, a charming young medical student (later pediatrician and psychotherapist). Their two kids were born in West Berlin during his residency and early career. Based on his successful early career and rapidly rising international reputation, he was promoted to medical director of St. Anna Children's Hospital in Vienna in 1980.

However, Helmut Gadner's influence on pediatric oncology development extended far beyond Austria's borders. The treatment protocols for acute leukemia he worked on during his early career at Charite Hospital in Berlin developed into a worldwide leading treatment concept under the branding "BFM" (abbreviation of the city names of the founding institutions, Berlin-Frankfurt-Münster). Upon his move to Vienna, he took the leadership for developing treatment for Langerhans cell histiocytosis (LCH), a rare disease, at that time known as "histiocytosis X" in the German-speaking countries. The success of his trials DAL-HX-83 and DAL-HX-90 and his international recognition entitled him to establish an international study center in Vienna for executing the LCH clinical trials of the Histiocyte Society and to shape the global clinical research for this disease for more than two decades [2-4].

His innovative thinking and foresight made him an internationally renowned leader. Gadner early recognized that research is vital to improving cures. With Dr. Erwin Senoner (the father of an affected child) and others, he founded St. Anna Children's Cancer Research Institute (St. Anna CCRI) in 1988, entirely funded by private donations. Under his thoughtful leadership and vision, St. Anna CCRI and St. Anna Children's Hospital grew in symbiotic relation to a worldwide acclaimed comprehensive center for research and innovative treatment for children and adolescents with cancer [5].

The authors of this review were blessed to be among Helmut Gadner's mentees and witness the national and international impact of his late career. For example, the current global standards for managing pediatric acute lymphoblastic leukemia, neuroblastoma, and LCH carry footprints of Helmut Gadner's research, the scientific output of his mentees, and the institutions he led.

Dr. Gadner set milestones in the development of pediatric oncology, and we firmly believe that the next generation of pediatric oncologists should know and endure his inspiring legacy.

## Review

Early career

**Figure 1:**
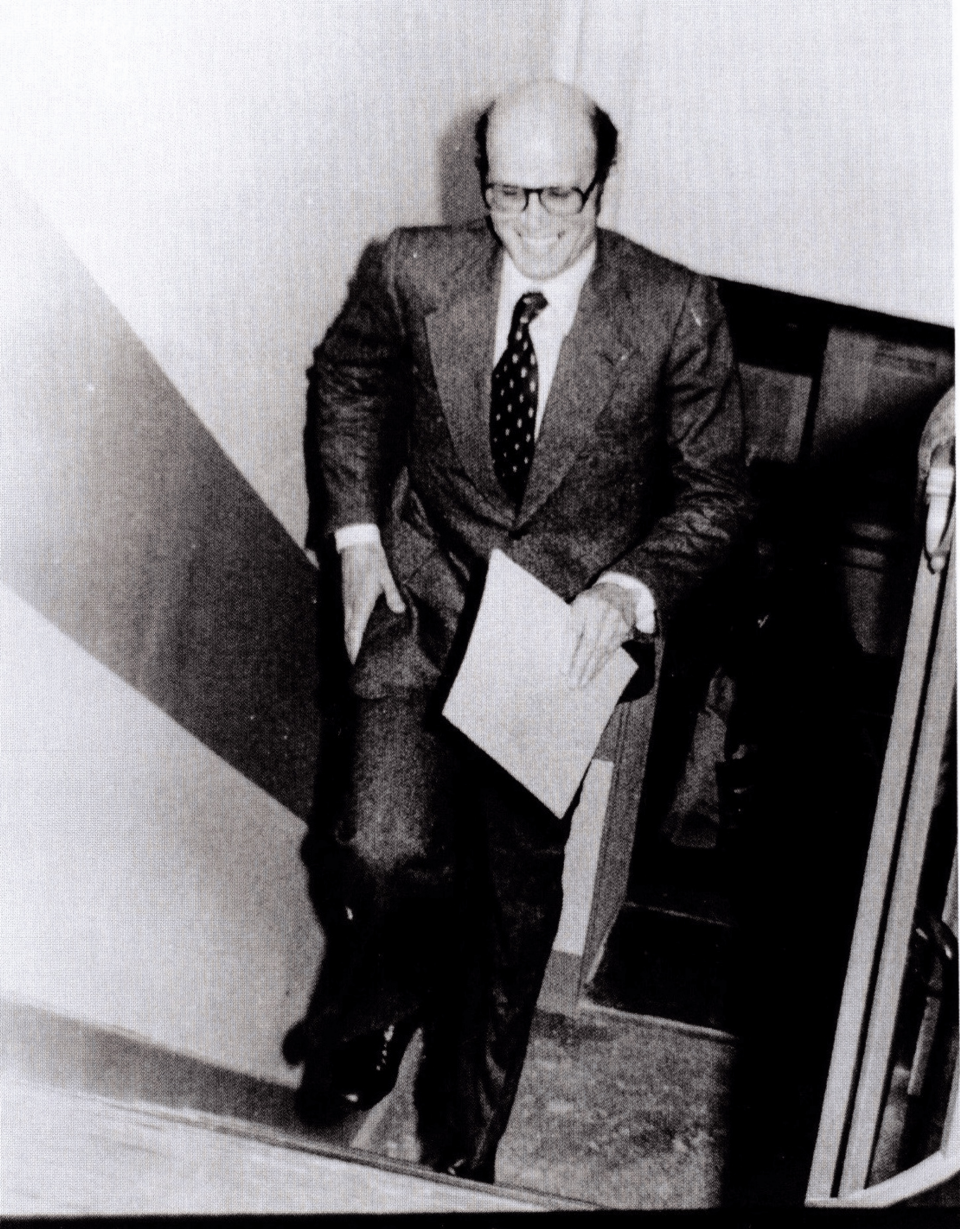
Dr. Helmut Gadner on the way to the stage to give the inaugural lecture at his academic promotion (Charité Hospital, West Berlin, 1978). Image credit: Family archive, provided by Johannes Gadner

Dr. Gadner's inspiration and choice for pediatric hematology and oncology during his residency in Berlin were greatly influenced by his mentor, Dr. Hansjörg Riehm, the father of the revolutionary BFM treatment concept (that is, early re-induction for pediatric lymphoblastic leukemia) [6-8]. In 1972, Dr. Gadner qualified as a senior physician, and in 1978, he was promoted to professor of pediatric hematology and oncology at the Charité University Hospital in West Berlin (Figure 1) [9].

During his early career in Germany, Helmut Gadner was part of the team designing and administrating the BFM treatment protocols. This early experience under Dr. Riehm's mentorship honed Gadner's expertise and positioned him as a future leader in the field, setting the stage for his later accolades and achievements in pediatric oncology [6].

**Figure 2:**
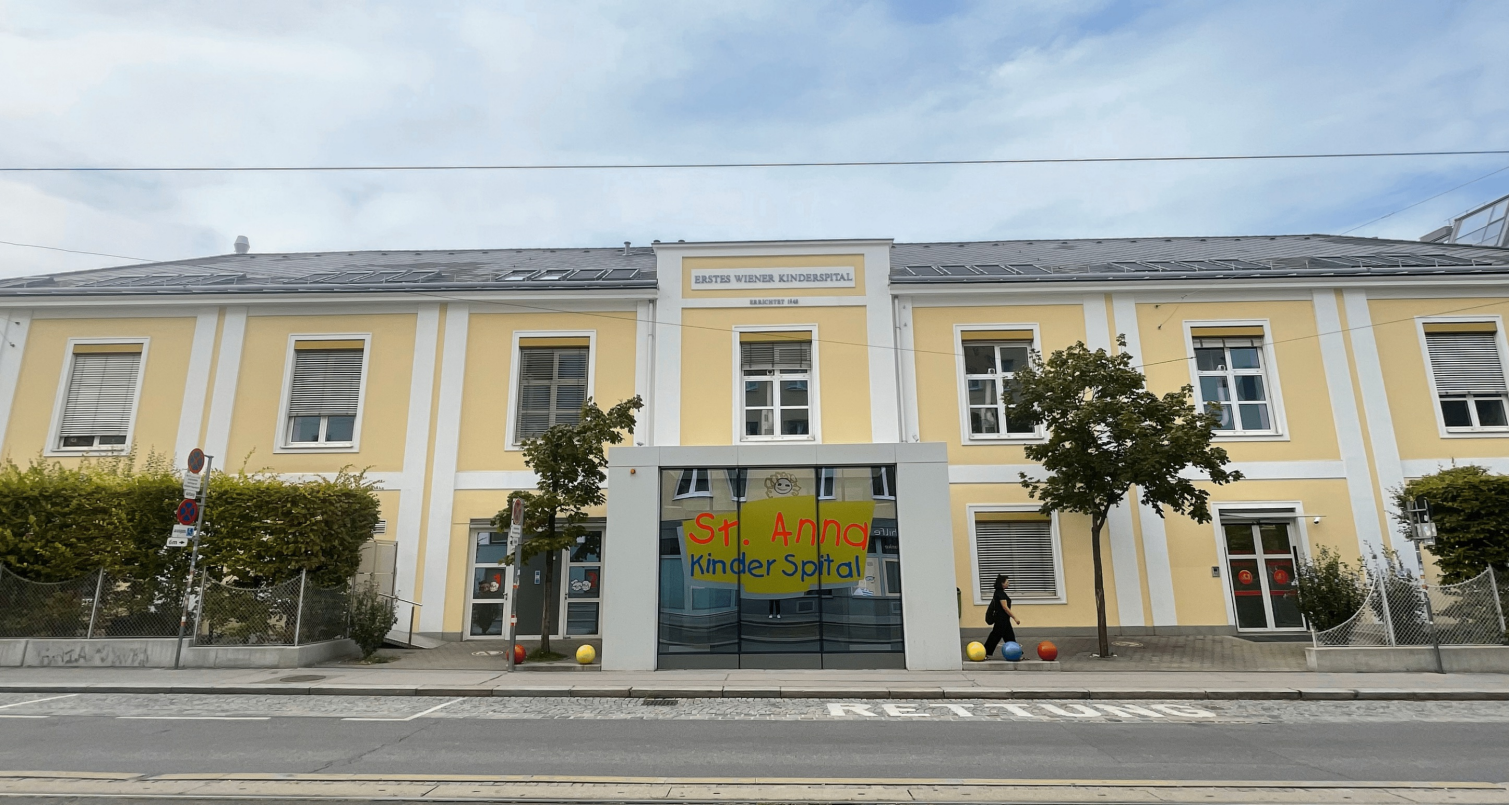
The historic front side of St. Anna Children's Hospital Image credit: Damian Dürrschnabel, 2024

Dr. Gadner was appointed medical director of St. Anna Children's Hospital in Vienna for his impressive research and growing international reputation. Under his leadership, this historical pediatric hospital (founded as the first pediatric hospital in Austria in 1837) was modernized and expanded to embrace a 24/7 emergency outpatient clinic, specialized outpatient clinics, and 125 patient beds for general pediatrics (four wards), pediatric hematology-oncology (two wards), a stem cell transplant unit, and an intensive care unit (Figure 2).

St. Anna Children's Hospital

From 1980 to 2010, Helmut Gadner served as the medical director of St. Anna Children's Hospital in Vienna and, in 1986, was awarded an honorary professorship at Vienna University. As the chief medical officer, Helmut Gadner was responsible for the entire hospital's patient care and strategic development. He initiated rebuilding and modernizing the hospital to meet international standards of pediatric care while preserving the historical part for administrative purposes and housing the emergency outpatient department. By his initiative, a stem cell transplantation unit was established.

There was not a single patient on the oncology ward or a seriously sick child in the general pediatric wards who was not seen by Dr. Gadner personally, and he was 24/7 available for advice to the medical staff. Moreover, Dr. Gadner was an excellent teacher. Especially his Monday evening lectures at the pediatric oncology ward at St. Anna Children's Hospital teaching were highly appreciated by his students, some of whom later also became leaders in pediatric oncology.

Dr. Gadner quickly gained national recognition as a brilliant pediatrician and dedicated children's advocate. His active professional contributions and social and political engagement for the well-being of Austrian children and adolescents culminated in his presidency of the Austrian Society of Pediatrics (ÖGKJ) from 1997 to 1999.

**Figure 3:**
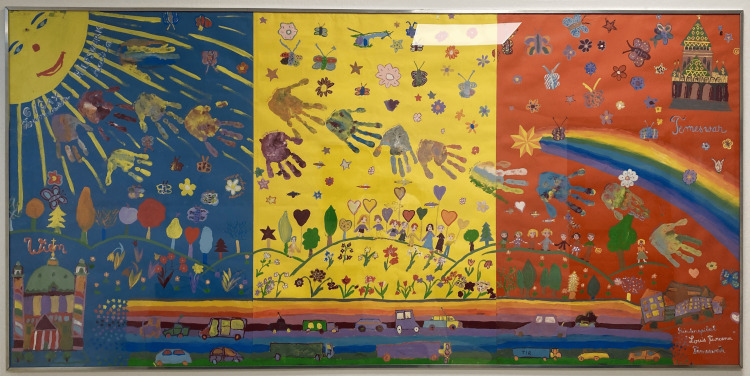
Painting of grateful children with cancer from Timisoara as an appreciation of Helmut Gadner's contributions to the local hospital Image credit: Leo Kager, 2024

Frequently receiving requests for advice in complex cases from other countries, Dr. Gadner generously shared his experience and advised colleagues worldwide. With his engagement, he made the advanced treatments established in St. Anna Children's Hospital accessible to kids from East European countries, thus saving many children's lives. He offered pro bono treatment (most often stem cell transplantation) in St. Anna Children's Hospital to children from Eastern Europe needing therapies not available in their home countries. Those personal experiences and his advisory roles in several non-governmental initiatives in countries of Eastern Europe and Central Asia enlightened him about the urgent need for improving care for children with cancer in those regions. With the dedicated support of the GIGAX foundation, he established training programs for physicians, nurses, psychologists, and scientists from those countries to spread knowledge, skills, and technology. Helmut Gadner traveled to Moscow to personally oversee and guide the first stem cell transplantation at the Federal Institute for Pediatric Hematology, Oncology, and Immunology in Moscow, performed by a team of physicians and nurses trained in Vienna. With financial support from the Austrian Government, personnel training through the GIGAX foundation, and the personal engagement and professional know-how of Helmut Gadner, the pediatric hematology-oncology department of Louis Turcanu Emergency Hospital for Children in Timisoara, Romania, was modernized to comply with international standards of care. A painting of grateful children with cancer from Timisoara at the entrance of his hospital office perpetuates his contributions to the local hospital and pediatric oncology in Romania (Figure 3).

Those and many other charitable activities of Helmut Gadner qualify him as a pioneer in the fight against inequalities in children with cancer. 

St. Anna Children's Cancer Research Institute

**Figure 4:**
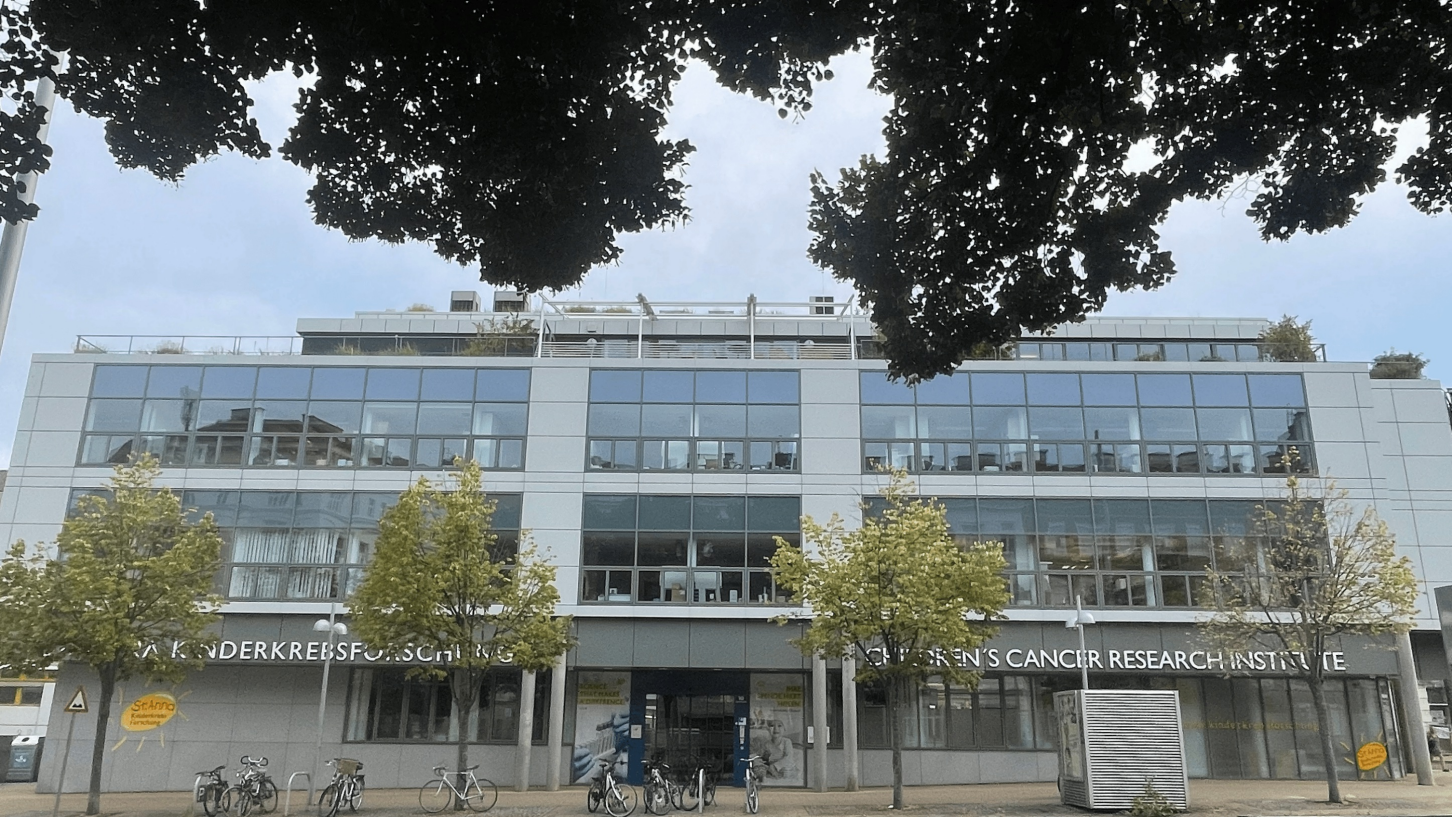
The current building of St. Anna Children's Cancer Research Institute at Zimmermannplatz 10, 1090 Vienna, opened in 2009. Image credit: Damian Dürrschnabel, 2024

**Figure 5:**
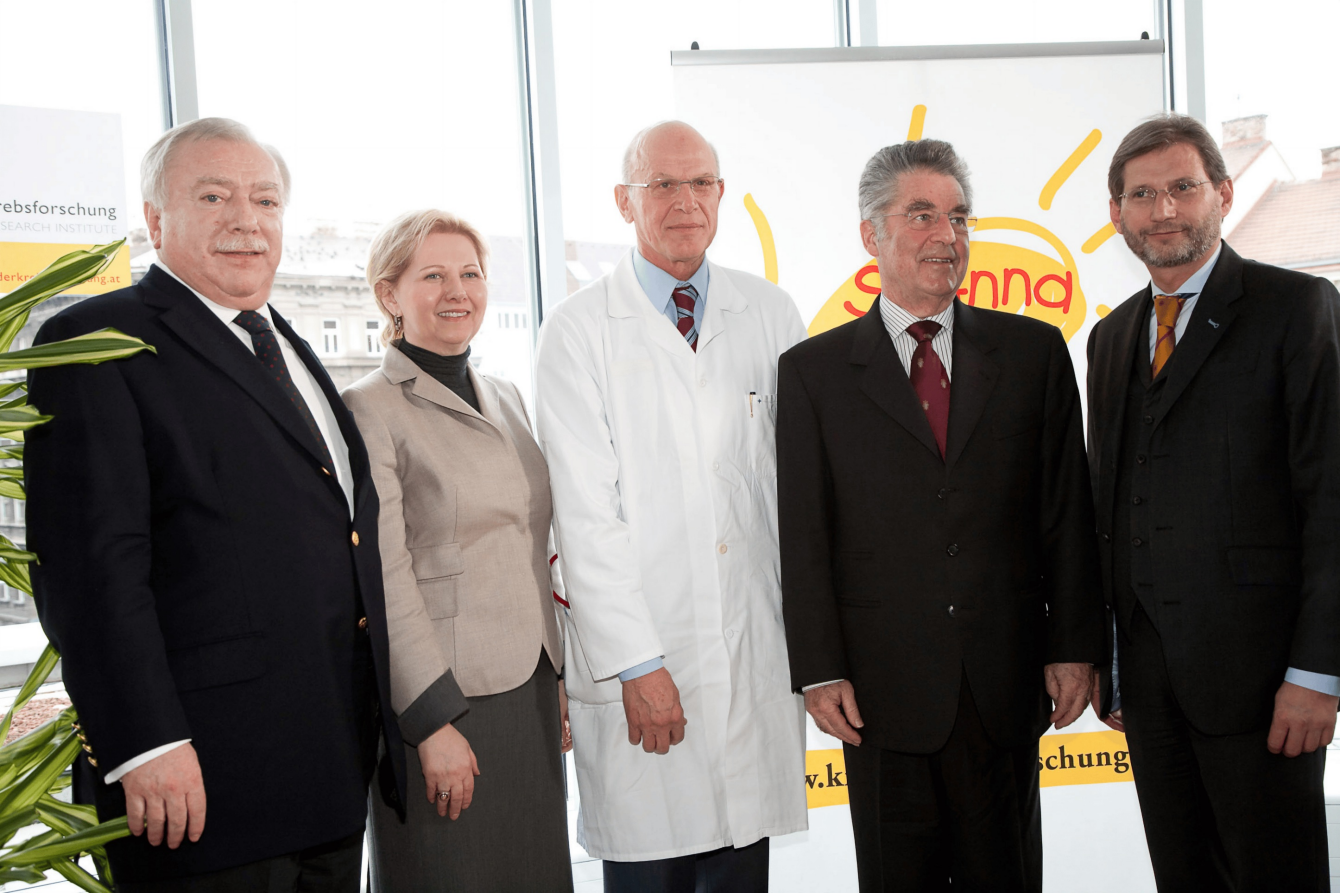
Opening ceremony of the new building of the CCRI in 2009 From left to right: Dr. M. Häupl (Vienna Mayor), B. Jank (Vienna Chamber of Commerce President), Helmut Gadner (St. Anna Children's Cancer Research Institute (CCRI) Director), Dr. H. Fischer (The President of Austria), and J. Hahn (Minister of Science). Image credit: With the permission of APA-Fotoservice/Ludwig Schedl, 2009

Soon after assuming the highly responsible hospital director and chief medical officer position at St. Anna Children's Hospital, Dr. Gadner realized that improving patient care mandates rigorous research from bench to bedside. With Dr. Erwin Senoner (the father of an affected child) and others, he founded in 1985 a charity, a private endeavor to raise awareness about pediatric cancer in the community and collect funds for independent research. In 1988, after three years of diligent conceptual work and fund collection, St. Anne CCRI was opened, initially operating five laboratory units in the attic of the historic hospital building. Dr. Gadner was the institute's director and a tireless strategic mind, leader, and mentor until his retirement in 2012 [5]. A historic milestone in the development of the CCRI was the construction of a dedicated building (Figure 4) next to St. Anna Children's Hospital, which opened in 2009. The president of Austria, accompanied by many prominent officials from the healthcare system, science, industry, and politics, attended the opening ceremony to pay tribute to this epochal event (Figure 5).

**Figure 6:**
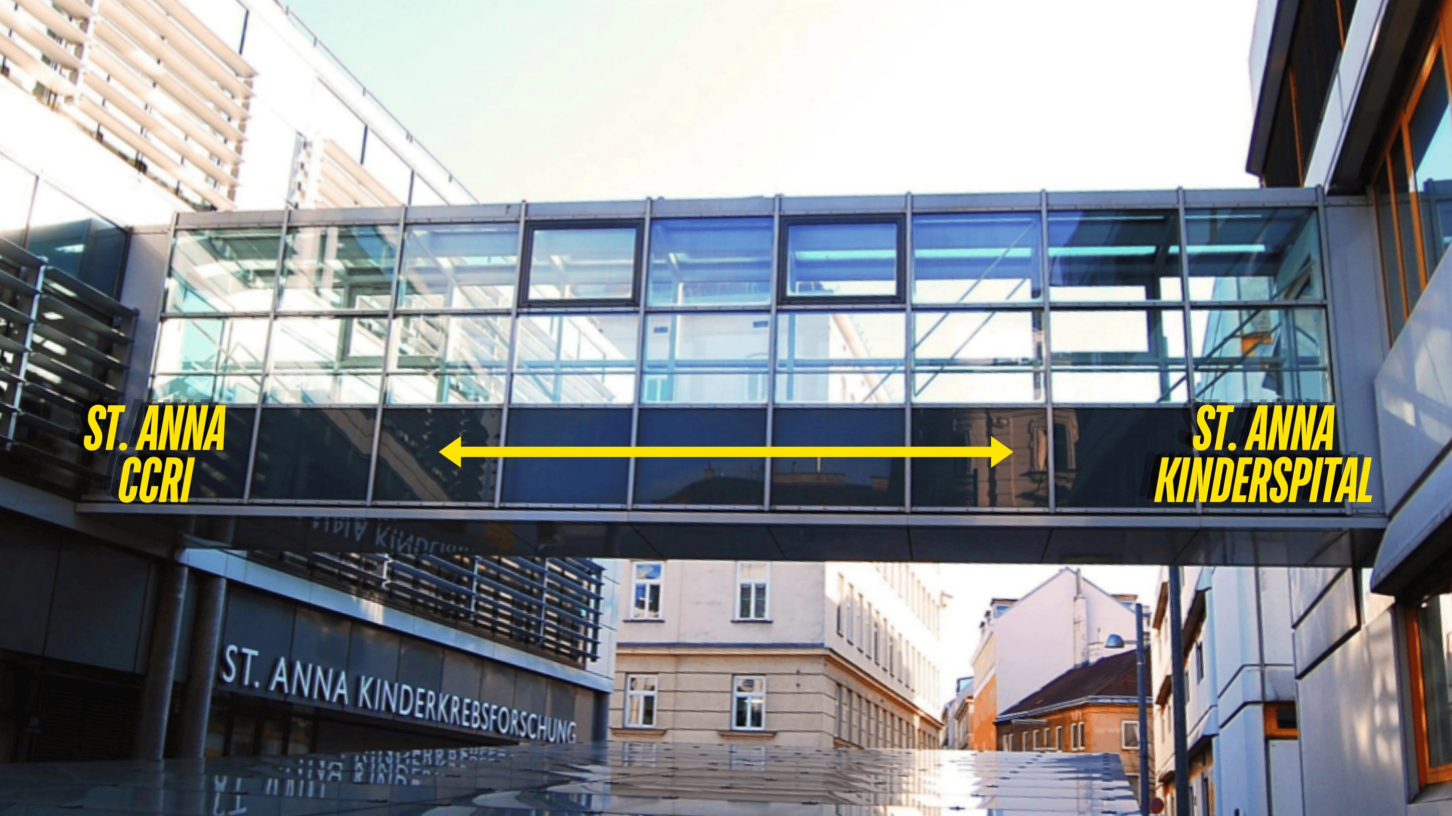
The bridge between CCRI and St. Anna Children's Hospital, symbolizing the coexistence and interdependence of research and quality patient care Image credit: Archive of the St. Anna Kinderkrebsforschung (arrow and direction indication by Lukas Minkov)

St. Anna CCRI, Gadner's enduring legacy, recently celebrated its 35^th^ anniversary. It operates 14 research groups with researchers from 36 nations actively engaged in global collaboration and various research fields, from basic molecular research to prospective international clinical trials. The bridge connecting St. Anna CCRI with St. Anna Children's Hospital, symbolizing the coexistence and interdependence of science and quality patient care, is a monument of Helmut Gadner's conviction and keeper of his legacy to the next generations of physicians and scientists (Figure 6).

International LCH Study Reference Center of the Histiocyte Society

Immediately after his move to Vienna, Helmut Gadner was appointed to lead a working group on LCH encompassing interested physicians and institutions from Austria, Germany, Switzerland, and the Netherlands, named DAL-HX. Under his leadership, the group conducted two subsequent clinical trials, which led to improved outcomes in patients with multisystem LCH [10-13]. The international LCH-II, LCH-III, and the currently ongoing LCH-IV clinical trials incorporated and further developed the treatment backbone Gadner successfully invented in his early studies.

Helmut Gadner attended the inaugural meeting in Philadelphia, PA, USA, which led to the foundation of the Histiocyte Society, an international non-profit organization of physicians and scientists committed to researching and improving care for patients with histiocytic disorders. Dr. Gadner has served on several standing committees and was president of the Histiocyte Society from 1992 to 1996. He was appointed to lead the LCH Study Group of the Histiocyte Society, which, during the two decades of his leadership, published several white papers paving the way for international collaboration and consequently designed and conducted the international prospective clinical studies LCH I-III [2-4, 14-16]. The knowledge and lessons from those studies form the basis of the current treatment standard for multisystem LCH in children and adolescents [17]. Guided by the unmet need for understanding LCH pathobiology and rational cure, Helmut Gadner encouraged and supported establishing a dedicated group to study LCH biology at the CCRI. Under the leadership of the current medical director of St. Anna Children's Hospital, Professor Caroline Hutter, this group has made some pivotal discoveries [18-20].

Legacy and impact

Helmut Gadner's impact on pediatric hematology and oncology on national and international levels is multifaceted, and full coverage (even just a list of his contributions) would go beyond the limits of a single review paper. The numerous prestigious awards throughout his career are an eloquent testimonial of his significant scientific contributions to medicine, particularly to the fields of pediatric oncology and hematology. His achievements are as follows: Grand Decoration of Honor for Services to the Republic of Austria (This award is one of the highest civilian honors in Austria, awarded for meritorious achievements in various fields, including healthcare and science); Austrian Cross of Honor for Science and Art, First Class (This high distinction from Austria recognizes outstanding achievements in science and art); Austrian Champion in European Research (The Austrian Research Promotion Agency honors his leadership in European scientific research); Hansjörg Riehm Prize for Excellence in Pediatric Hematology and Oncology (awarded for outstanding contributions specifically within the field of pediatric hematology and oncology); Lifetime Achievement Award of the European Society for Pediatric Oncology (SIOP Europe) 2009; Histiocyte Society Golden Pin recipient for 2008 (The Golden Pin Award of the Histiocyte Society, in collaboration with the Histiocytosis Association, is a prestigious recognition given to individuals who have demonstrated outstanding contributions to the field of histiocytic disorders); Fellow of the Royal College of Physicians and Surgeons of Glasgow (This fellowship recognizes professional achievement and commitment to ongoing learning and development in the medical field); Honorary member of the German Society of Pediatric Hematology and Oncology (GPOH); Honorary member of the Histiocyte Society.

**Figure 7:**
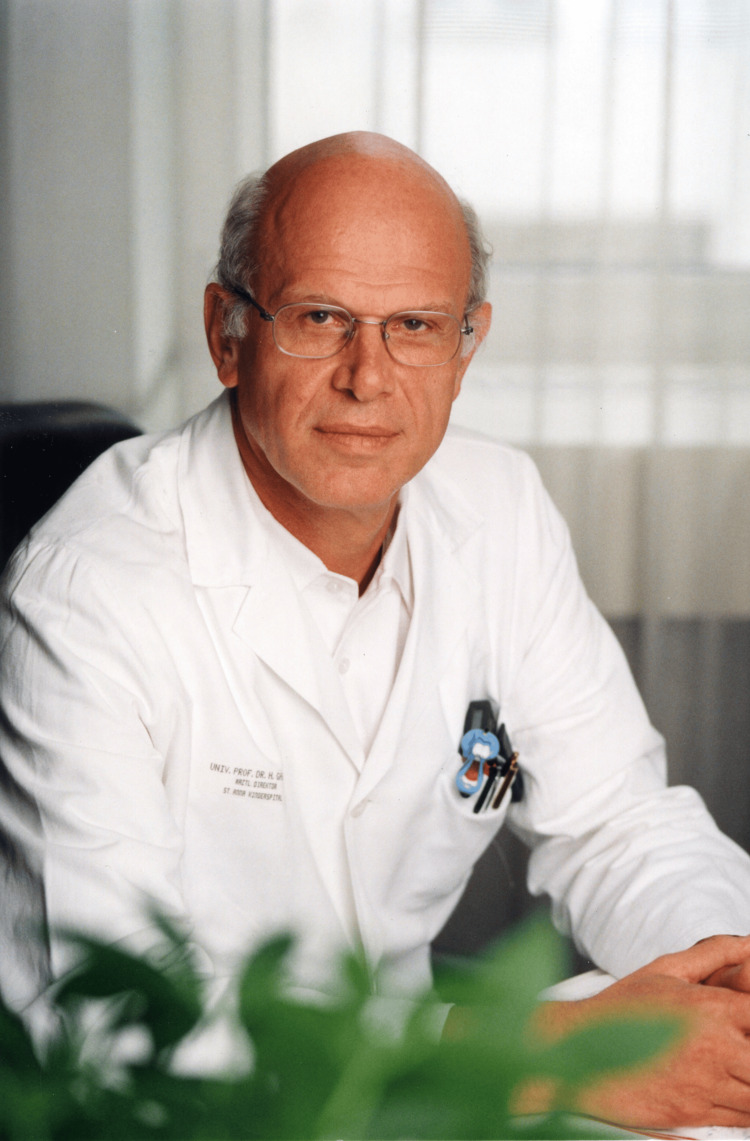
Professor Helmut Gadner in his St. Anna Children's Hospital office Image credit: From the archives of St. Anna Children's Hospital (provided by David Steiner)

These accolades highlight Dr. Gadner's influential role in advancing medical research and treatment methodologies for childhood cancers and his respected status within national and international medical communities. For his scientific achievements and publication treatise (473 papers in PubMed as of August 4, 2024), Helmut Gadner was awarded 2000 as a Full Member of the Division of Mathematics and Natural Sciences of the Austrian Academy of Sciences [9]. Most importantly, Helmut Gadner was beloved by his patients, their parents, and colleagues (Figure 7).

## Conclusions

Helmut Gadner engaged in developing the BFM critical trials for acute lymphoblastic leukemia during his early professional career. His early scientific contributions to global pediatric hematology and oncology were instrumental in establishing protocols that have saved countless young lives worldwide. Dr. Gadner’s engagement in the 1990s against inequalities in pediatric cancer in the former socialist countries of Eastern Europe and Central Asia was visionary, and the fruits of his pioneer work continue to save lives in those countries.

Without a doubt, the most incredible and enduring legacy of Helmut Gadner is the foundation and his leadership of St. Anna CCRI, one of the world's most productive and successful research facilities for pediatric cancer.
